# Prostate cancer and elective nodal radiation therapy for cN0 and pN0—a never ending story? Recommendations from the prostate cancer expert panel of the German Society of Radiation Oncology (DEGRO)

**DOI:** 10.1007/s00066-023-02193-4

**Published:** 2024-01-25

**Authors:** S. A. Koerber, S. Höcht, D. Aebersold, C. Albrecht, D. Boehmer, U. Ganswindt, N.-S. Schmidt-Hegemann, T. Hölscher, A.-C. Mueller, P. Niehoff, J. C. Peeken, M. Pinkawa, B. Polat, S. K. B. Spohn, F. Wolf, C. Zamboglou, D. Zips, T. Wiegel

**Affiliations:** 1 Department of Radiation Oncology, Barmherzige Brüder Hospital Regensburg, Prüfeninger Straße 86, 93049 Regensburg, Germany; 2 Department of Radiation Oncology, Heidelberg University Hospital, Im Neuenheimer Feld 400, 69120 Heidelberg, Germany; 3 Department of Radiation Oncology, Ernst von Bergmann Hospital Potsdam, Charlottenstraße 72, 14467 Potsdam, Germany; 4 Department of Radiation Oncology, Inselspital—Bern University Hospital, University of Bern, Freiburgstraße 4, 3010 Bern, Switzerland; 5 Nordstrahl Radiation Oncology Unit, Nürnberg North Hospital, Prof.-Ernst-Nathan-Str. 1, 90149 Nürnberg, Germany; 6 Department of Radiation Oncology, University Hospital Berlin, Augustenburger Platz 1, 13353 Berlin, Germany; 7 Department of Radiation Oncology, University Hospital Innsbruck, Anichstraße 35, 6020 Innsbruck, Austria; 8 Department of Radiation Oncology, University Hospital, LMU Munich, Marchioninistraße 15, 81377 Munich, Germany; 9 Department of Radiotherapy and Radiation Oncology, Faculty of Medicine and University Hospital Carl Gustav Carus, TU Dresden, Fiedlerstraße 19, 01307 Dresden, Germany; 10 Department of Radiation Oncology, RKH Hospital Ludwigsburg, Posilipostraße 4, 71640 Ludwigsburg, Germany; 11 Department of Radiation Oncology, Sana Hospital Offenbach, Starkenburgring 66, 63069 Offenbach, Germany; 12 Department of Radiation Oncology, Technische Universität München, Ismaninger Straße 22, 81675 Munich, Germany; 13 Department of Radiation Oncology, Robert Janker Klinik, Villenstraße 8, 53129 Bonn, Germany; 14 Department of Radiation Oncology, University Hospital Würzburg, Josef-Schneider-Straße 11, 97080 Würzburg, Germany; 15 Department of Radiation Oncology, University Hospital Freiburg, Robert-Koch-Straße 3, 79106 Freiburg, Germany; 16 Department of Radiation Oncology, Paracelsus Medical University of Salzburg, Müllner Hauptstraße 48, 5020 Salzburg, Austria; 17 German Oncology Center, 1, Nikis Avenue, Agios Athanasios, 4108 Limassol, Cyprus; 18 Department of Radiation Oncology, University Hospital Ulm, Albert-Einstein-Allee 23, 89081 Ulm, Germany

**Keywords:** ENI, Pelvic nodal treatment, Prostate carcinoma, Radiotherapy, Pelvic irradiation

## Abstract

For prostate cancer, the role of elective nodal irradiation (ENI) for cN0 or pN0 patients has been under discussion for years. Considering the recent publications of randomized controlled trials, the prostate cancer expert panel of the German Society of Radiation Oncology (DEGRO) aimed to discuss and summarize the current literature. Modern trials have been recently published for both treatment-naïve patients (POP-RT trial) and patients after surgery (SPPORT trial). Although there are more reliable data to date, we identified several limitations currently complicating the definitions of general recommendations. For patients with cN0 (conventional or PSMA-PET staging) undergoing definitive radiotherapy, only men with high-risk factors for nodal involvement (e.g., cT3a, GS ≥ 8, PSA ≥ 20 ng/ml) seem to benefit from ENI. For biochemical relapse in the postoperative situation (pN0) and no PSMA imaging, ENI may be added to patients with risk factors according to the SPPORT trial (e.g., GS ≥ 8; PSA > 0.7 ng/ml). If PSMA-PET/CT is negative, ENI may be offered for selected men with high-risk factors as an individual treatment approach.

## Introduction

Elective nodal irradiation (ENI) is performed for many tumors; however, there is still a debate regarding its oncological benefit for several malignancies. For many years, elective nodes have been included unintentionally in the target volume due to technical reasons. Nowadays, intensity-modulated radiation therapy (IMRT) is commonly used, enabling a more targeted therapeutic approach [1, 2]. Numerous trials focus on de-escalation strategies for radiation therapy volumes to reduce side effects [3]. Moreover, the growing use of hypofractionation and stereotactic body radiation therapy (SBRT) with high single doses again raises the question about the size of radiation fields for several tumors [4, 5].

One of these malignancies is prostate cancer, where a long-term discussion about ENI is still active.

Large studies demonstrated inconsistent results for both the primary and the postoperative setting by including pelvic nodes for N0 cancer patients. Several prospective trials have been published during recent years; however, a common recommendation is lacking to date [6]. Therefore, the current manuscript aimed to discuss and summarize the current literature and tries to give some clinical recommendations for ENI in the primary and postoperative setting.

## Definitive radiation therapy for prostate cancer with elective nodal irradiation (cN0)—current literature

For the primary setting, there is a large heterogeneity within randomized trials evaluating the role of radiation therapy and androgen deprivation therapy (ADT) for prostate cancer with risk factors. While several trials have included elective pelvic nodes [7, 8], some have not [9, 10]. In total, four large randomized trials with long-term follow-up focusing on the role of ENI are available [11–14]. Updated results of the GETUG-01 and RTOG 9413 trials were published in 2016 and 2018, respectively [15, 16]. In the French trial, there was a lack of an oncological benefit for treatment-naïve men (cN0) who received whole-pelvis radiation therapy (WPRT; 46 Gy; 23 fractions; prostate boost: 66–70 Gy) compared to prostate-only radiation therapy (PORT; 66–70 Gy; 1.8–2.0 Gy per fraction) after a median follow-up of almost 9 years. The authors observed no statistically significant difference in event-free survival (primary endpoint) or overall survival for the entire cohort of 446 patients [15]. RTOG 9413 enrolled 1322 men with cN0 prostate cancer after conventional staging and a risk for nodal metastases of more than 15% according to the Roach formula. The trial with a 2 × 2 design treated patients with neoadjuvant vs. adjuvant hormonal treatment (HT) and WPRT (50.4 Gy, 28 fractions; prostate boost: 70 Gy) vs. PORT (70.2 Gy, 39 fractions). After a median follow-up of 108 months, 10-year estimates of progression-free survival (primary endpoint) were low for all treatment arms. The best PFS was observed in the PORT plus adjuvant hormonal therapy and in the WPRT plus neoadjuvant hormonal therapy group. However, the large field size in combination with neoadjuvant HT was associated with a significantly higher rate of late grade 3+ gastrointestinal toxicity [16]. In both trials, no modern techniques such as IMRT were used.

In 2019, first results of a randomized controlled phase II trial were published, where IMRT and dose-escalated radiation therapy were standardly used (PIVOTAL). All patients (*n* = 124) were staged cN0 and had a risk for nodal metastases of more than 30% according to the Roach formula. WPRT was performed with a total dose of 60 Gy (pelvis) and 74 Gy (prostate) in 37 fractions, patients in the PORT group received 74 Gy in 37 fractions. With a median follow-up of 24 months, the authors concluded that high-dose WPRT can be delivered with a modest side effect profile; outcome data are, however, still lacking [17]. Oncological results were provided by a recently published study from India. The POP-RT trial included 224 men with biopsy-proven prostate cancer and a risk of nodal involvement of more than 20% (Roach formula). All men were staged as cN0 using modern PSMA-PET/CT (80%), received adequate ADT, and were randomized 1:1 to WPRT or PORT. Patients received luteinizing hormone-releasing hormone (LHRH) analogs for 2 years; however, bilateral orchiectomy (18.9%) was also allowed. For this cohort, biochemical failure-free survival (primary endpoint), disease-free survival, and distant MFS were significantly higher for the WPRT group (50.0 Gy/68.0 Gy; 25 fractions) than for the PORT group (68 Gy; 25 fractions) after a median follow-up of 68 months. The authors observed no difference with regard to overall survival [18].

## Definitive radiation therapy for prostate cancer with elective nodal irradiation (cN0)—interpretation of trial results

Only the POP-RT trial has demonstrated an oncological benefit for treatment-naïve men with prostate cancer undergoing WPRT so far (Table 1). With longer follow-up, also a benefit for overall survival can be expected considering that improved distant metastasis-free survival was found to be a strong surrogate for overall survival in prostate cancer patients [19]. Comparing this trial with results obtained from GETUG-01 and RTOG 9413, some reasons can be identified which might explain this difference: while doses of only 66–70 Gy to the prostate were applied in the two trials, dose-escalated radiation therapy was performed in the POP-RT trial, resulting in improved local control [13, 14, 18]. Data from prospective and retrospective trials indicate that local control is a significant parameter if outcome is affected by WPRT or not [20–22]. Second, patients enrolled in the POP-RT trial were at high or very high risk. Almost 50% of all patients had a calculated risk for nodal metastases exceeding 40% and half of the cohort was classified as Gleason group 4 or 5 [18]. Thus, a very large proportion of men had a substantial risk for (microscopic and PSMA-negative) nodal metastasis and probably benefited the most from WPRT. Moreover, target volume delineation for WPRT was not consistent for the trials. While the common iliac nodes were consistently included in the target volume in the POP-RT trial, no sufficient coverage was obtained for the common iliac nodes, parts of the external iliac nodes, and presacral nodes in the GETUG-01 trial [15, 18]. Also for RTOG 9413, a relatively small WPRT volume was used (upper boarder: L5–S1 interspace) [16]. Smaller target volumes might have caused a higher rate of nodal failure occurring outside of “historical boundaries” [23, 24]. Thus, older trials should be interpreted with caution and target volume delineation should be performed according to international guidelines.

The Canadian prospective two-arm PCS5 trial evaluated the role of normofractionated with hypofractionated WPRT (61% IMRT) for patients with prostate cancer and high-risk features. The authors reported excellent results with regard to toxicity and presumably oncological endpoints, with no significant differences [25]. In terms of rising hypofractionated regimes, the full publication, including outcome data, is highly awaited [26]. While also expecting results from RTOG 0924 and the PIVOTALboost trial [23, 27], the current literature suggest a benefit for ENI in cN0 prostate cancer patients with a very high risk of positive pelvic nodes, although primary staging was performed with PSMA-PET/CT. This can be caused by the limited sensitivity of 68 Gy leading to a lack of detection of small nodal metastases [28]. In the POP-RT trial, a biological dose of 50 Gy was applied to the pelvic nodes, leading to a low rate of in-field relapses. Moreover, long-term ADT (≥ 24 months) was administered [18], which seems to positively affect outcome data again. The use of IMRT is able to avoid a significant increase in severe toxicity [17, 18, 29]. However, the POP-RT trial also has some limitations, including a relatively large rate of patients receiving permanent castration (orchiectomy) and the small study cohort. Moreover, the trial may be underpowered due to the low rate of events (36) observed after a study period of 9 years [18]. Therefore, the trial results should be interpreted with a certain caution.

Considering the current literature, there is no general recommendation for ENI for patients (cN0) undergoing definitive radiation therapy. However, some men with a high risk for nodal involvement (e.g., cT3a, GS ≥ 8, PSA ≥ 20 ng/ml) may benefit from the addition of pelvic lymph node treatment. To a lesser extent, this also applies for treatment-naïve patients with primary PSMA staging.

## Postoperative radiation therapy for prostate cancer with elective nodal irradiation (pN0)—current literature

While some evidence exists for pelvic (adjuvant) irradiation for men with pN1 in the postoperative setting [30–33], there are no reliable data for prostate cancer patients without nodal lesions so far. Retrospective trials suggest a potential benefit for WPRT +/− ADT with regard to biochemical progression-free survival [34, 35]. A propensity score-matching analysis with data from 191 patients identified WPRT as an independent factor for bPFS compared to prostate bed radiation therapy (PBRT) only [35]. Similar results were observed in a database analysis with 1861 patients, reporting a higher rate of biochemical failure for prostate bed irradiation (compared to WBRT) and foregoing ADT [34]. One prospective phase II study (McGill 0913) combining long-term ADT and pelvic radiation therapy in the postoperative management of high-risk prostate cancer patients concluded that the regime appeared promising and tolerable. However, the cohort was small (*n* = 46) and the proportion of pN0 candidates was limited [36].

Recently, a large prospective randomized multicenter trial was published evaluating the role of ADT and pelvic lymph node treatment in the postoperative setting: the RTOG 0543 SPPORT trial (primary endpoint: freedom from progression) enrolled 1792 men with PSA relapse after surgery and randomly assigned to prostate bed radiation therapy alone (arm 1), PBRT with ADT (arm 2), and PBRT plus ENI and ADT (arm 3) [37]. The prostate bed was treated with doses from 64.8 to 70.2 Gy (single dose 1.8 Gy) and ENI was performed with a total dose of 45.0 Gy (single dose 1.8 Gy). Patients received ADT for 4–6 months, an IMRT technique (87.2%) was used in all three treatment approaches. After a median follow-up of 8.2 years, the highest freedom-from-progression and acute ≥ grade 2 toxicity rates were obtained for arm 3 (87.4%; 44%) compared to arm 2 (81.3%; 36%) and arm 1 (70.9%; 18%). No significant difference regarding overall survival and late toxicity except for hematological side effects (arm 3) was observed for the entire cohort [37]. Thus, the authors concluded that ADT and the extension of the standard target volume after surgery may have a noticeable impact on the outcome of post-prostatectomy patients [37].

## Postoperative radiation therapy for prostate cancer with elective nodal irradiation (pN0)—interpretation of trial results

The SPPORT trial is a large randomized trial with acceptable follow-up and the use of modern radiation therapy techniques (Table 2). The trial demonstrated convincing data at first glance, suggesting a high benefit for short-term ADT and ENI for men with PSA relapse after surgery. However, some relevant limitations challenge the interpretation of the current trial: first, more than one third of all patients received no lymphadenectomy and the median number of resected lymph nodes was only six, concealing a relevant number of patients with positive nodes. Moreover, the definition of the primary endpoint is incomprehensible considering that the Phoenix criteria are not validated for the postoperative setting. Finally, the lack of PSMA-PET/CT before the start of radiation therapy is a major limitation in the era of PSMA-PET-guided salvage radiotherapy [38–41]. Thus, a potentially large number of positive pelvic nodes may have remained undetected and were not accessible for local treatment approaches. Similar trials, like the prospective randomized GETUG-AFU-16 or RTOG 9601, both evaluating the role of concomitant systemic therapy in patients with postoperative radiation therapy, included only one type of ADT (goserelin/bicalutamide) [42, 43].

Therefore, many comments with justifiable criticism arose after publication of the SPPORT trial [44–47]. For patients undergoing salvage radiation therapy, evaluation of the need for ADT according to risk or unfavorable prognostic factors (pre-salvage PSA levels > 0.6 ng/ml, Gleason score > 7, PSA doubling time < 12 months) is supposed to be the first and most important step [48–50]. The SPPORT trial was able to confirm the results obtained from GETUG-AFU-16, where short-term ADT improved freedom from progression [37, 43]. In the current trial, no significant difference occurred between 4 months compared to 6 months ADT [37]. While the duration and the type of antihormonal therapy is still under discussion, first results of the three-arm randomized RADICALS-HD trial were presented at the 2022 ESMO congress. The authors reported that short-term ADT (6 months) compared to no ADT improved time to salvage ADT but not MFS [51]. However, long-term ADT (24 months) compared to short-term ADT was able to improve both MFS and time to salvage ADT [51]. The full publication of the trial is highly awaited and will provide reliable data with regard to systemic therapy.

In conclusion, for postoperative patients and no available PSMA-PET/CT, ENI may be performed for men with high-risk factors (e.g., Gleason score ≥ 8; PSA > 0.7 ng/ml) according to the SPPORT trial. For patients who underwent PSMA imaging without positive nodes, WPRT may be offered as an individual treatment approach when numerous risk factors are present (e.g., Gleason score ≥ 8, short PSA doubling time < 6 months, pT3-4). However, reliable data for this situation are lacking so far.

## Conclusion

For treatment-naïve patients with prostate cancer, we are not able to recognize a general recommendation for ENI considering current literature and salvage treatment options (e.g., radiation therapy of nodal failure). However, normofractionated (45.0–54.0 Gy) WPRT may be offered to patients with high-risk factors according to the POP-RT trial (e.g., cT3a, GS ≥ 8, PSA ≥ 20 ng/ml), regardless of the presence of primary PSMA imaging.

For the postoperative setting, two scenarios should be differentiated:

1. Patients with PSA relapse and no PSMA-PET/CT: considering data obtained from the SPPORT trial, ENI may be offered to patients with high-risk factors (e.g., Gleason score ≥ 8; PSA > 0.7 ng/ml). In this context, some attention should also be devoted to the extent of lymphadenectomy.
2. Patients with PSA relapse who underwent PSMA-PET/CT (without positive nodes): there is a lack of reliable data so far. However, WPRT may be performed for selected men with high-risk factors (e.g., Gleason score ≥ 8, short PSA doubling time < 6 months, pT3-4).

Overall, patients should be informed about an increased risk of toxicity.

**Table 1:** Overview of prospective trials with regard to ENI for treatment-naïve patients (cN0)

Trial name	Trial design	Number of patients	WPRT dose (total dose/single dose)	Radiation dose prostate (total dose/single dose)	ADT	Results (WPRT vs. PORT)
GETUG-01 [14, 15]	RCT Phase III	446	46/2 Gy	66–70/1.8–2 Gy	6 months^b^	10-year EFS 77.2% vs. 62.5% (n. s.); no OS difference
RTOG 9413 [13, 16]	RCT Phase III	1322	50.4/1.8 Gy	70.2/1.8 Gy	4 months (2 months NHT) or 4 months (AHT)	10-year PFS 28.4% (WPRT + NHT) vs. 23.5% (PORT + NHT) vs. 19.4% (WPRT + AHT) vs. 30.2% (PORT + AHT)
PIVOTAL [17]	2‑arm Phase II	124	60 Gy/1.62 Gy	74 Gy/2 Gy	Variable	At 2 years: G2 + GI toxicity 24.0% vs. 16.9%; G2+ bladder toxicity 5.6% vs. 5.1%
POP-RT [18]	RCT Phase III	224	50/2 Gy	68/2.72 Gy	2 years/orchiectomy	5‑year bFFS 95 vs. 81%; 5‑year DFS 90 vs. 77%; 5‑year dMFS 96 vs. 89%
RTOG 0924 [22]	RCT Phase III	2592	45/1.8 Gy	79.2/1.8 Gy or brachy boost	6 or 32 months	Active
PIVOTALboost [27]	RCT Phase III	1952	47/2.35 Gy	37.5/2.5 Gy + HDR-boost or 42/2.1 Gy + HDR-boost or 60/3 Gy +/− focal boost	6–12 months^a^/2–3 years^b^	Active

*ADT* androgen deprivation therapy, *AHT* adjuvant hormonal therapy, *NHT* neoadjuvant hormonal therapy, *RCT* randomized controlled trial, *WPRT* whole-pelvis radiation therapy

^a^for intermediate-risk prostate cancer

^b^only for high-risk prostate cancer

**Table 2:** Overview of prospective trials with regard to ENI for pN0 patients and PSA relapse after surgery

Trial name	Trial design	Number of patients	WPRT dose (total dose/single dose)	Radiation dose prostate bed (total dose/single dose)	ADT	Results
McGill 0913 [36]	Single arm Phase II	46	44/2 Gy	66/2 Gy	24 months	5‑year PFS 78%; low toxicity rates
RTOG 0543 SPPORT [37]	RCT Phase III	1792	45/1.8 Gy	64.8–70.2/1.8 Gy	None (arm 1) or 4–6 months (arm 2 + 3)	5‑year FFP 87 vs. 81 vs. 71%; no OS difference

*ADT* androgen deprivation therapy, *FFP* freedom from progression, *OS* overall survival, *PFS* progression-free survival, *RCT* randomized controlled trial, *WPRT* whole pelvis radiation therapy

## Abbreviations

ADT: Androgen-deprivation therapy
CT: Computed tomography
ENI: Elective nodal irradiation
GS: Gleason score
HT: Hormonal treatment
IMRT: Intensity-modulated radiation therapy
LHRH: Luteinizing hormone-releasing hormone
MFS: Metastasis-free survival
PBRT: Prostate bed radiation therapy
PET: Positron-emission tomography
PFS: Progression-free survival
PORT: Prostate-only radiation therapy
PSA: Prostate-specific antigen
PSMA: Prostate-specific membrane antigen
SBRT: Stereotactic body radiation therapy
WPRT: Whole-pelvis radiation therapy
